# FGF21 induced by carbon monoxide mediates metabolic homeostasis *via* the PERK/ATF4 pathway

**DOI:** 10.1096/fj.201700709RR

**Published:** 2018-01-08

**Authors:** Yeonsoo Joe, Sena Kim, Hyo Jeong Kim, Jeongmin Park, Yingqing Chen, Hyeok-Jun Park, Seung-Joo Jekal, Stefan W. Ryter, Uh Hyun Kim, Hun Taeg Chung

**Affiliations:** * Meta-Inflammation Research Institute of Basic Research, School of Biological Sciences, University of Ulsan, Ulsan, South Korea;; † Wonkwang Health Science University, Iksan, Jeonbuk, South Korea;; ‡ Division of Pulmonary and Critical Care Medicine, Joan and Sanford I. Weill Department of Medicine, Weill Cornell Medicine, New York, New York, USA; and; § National Creative Research Laboratory for Ca^2+^ Signaling Network, Chonbuk National University Medical School, Jeonju, South Korea

**Keywords:** hepatic steatosis, metabolic disease, ER stress, ROS, thermogenic genes

## Abstract

The prevalence of metabolic diseases, including type 2 diabetes, obesity, and cardiovascular disease, has rapidly increased, yet the molecular mechanisms underlying the metabolic syndrome, a primary risk factor, remain incompletely understood. The small, gaseous molecule carbon monoxide (CO) has well-known anti-inflammatory, antiproliferative, and antiapoptotic effects in a variety of cellular- and tissue-injury models, whereas its potential effects on the complex pathways of metabolic disease remain unknown. We demonstrate here that CO can alleviate metabolic dysfunction *in vivo* and *in vitro*. We show that CO increased the expression and section of the fibroblast growth factor 21 (FGF21) in hepatocytes and liver. CO-stimulated PERK activation and enhanced the levels of FGF21 *via* the eIF2α–ATF4 signaling pathway. The induction of FGF21 by CO attenuated endoreticulum stress- or diet-induced, obesity-dependent hepatic steatosis. Moreover, CO inhalation lowered blood glucose levels, enhanced insulin sensitivity, and promoted energy expenditure by stimulating the emergence of beige adipose cells from white adipose cells. In conclusion, we suggest that CO acts as a potent inducer of FGF21 expression and that CO critically depends on FGF21 to regulate metabolic homeostasis.—Joe, Y., Kim, S., Kim, H. J., Park, J., Chen, Y., Park, H.-J., Jekal, S.-J., Ryter, S. W., Kim, U. H., Chung, H. T. FGF21 induced by carbon monoxide mediates metabolic homeostasis *via* the PERK/ATF4 pathway.

Metabolic syndrome and obesity are increasing in prevalence and contribute a burden on public health (1, 2). These conditions may promote the pathogenesis of various metabolic diseases, including glucose metabolism disorders, hypertension, and dyslipidemia, and can represent risk factors for type 2 diabetes and cardiovascular disease (3, 4). The development of insulin resistance may provide a major mechanism for the pathophysiology of the metabolic syndrome. Furthermore, metabolic disease can be associated with other underlying factors, such as nonalcoholic fatty liver disease (5), and mediators, such as proinflammatory cytokines (6) and adiponectin (7, 8). Leptin resistance has been suggested as an alternative mechanism of metabolic syndrome (9). Based on these various mechanisms, the targeting of metabolic homeostasis may represent a potential therapeutic strategy for metabolic syndrome.

Carbon monoxide (CO) is an endogenously produced product of heme oxygenase enzyme activity. When applied exogenously and at a low concentration, CO can provide pleiotropic cyto- and tissue-protective effects in various models of cellular or tissue injury involving anti-inflammatory, antiproliferative, and antiapoptotic effects (10, 11). Furthermore, chronic treatment with CO-releasing molecules (CORMs) increases metabolism, resulting in weight loss (12, 13), and increases mitochondrial biogenesis and function (14, 15). CO may mediate metabolic homeostasis *via* alteration of mitochondrial function, lipid metabolism, and inflammation. Furthermore, CO can protect cellular functions and preserve homeostasis under stress, through the modulation of autophagy, a lysosome-dependent mechanism for the turnover of cellular substrates, such as lipids, protein aggregates, and damaged organelles (16).

Although low CO levels promote the activation of molecular and cellular pathways involved in metabolic homeostasis, high doses of CO can cause acute clinical effects, including nausea, dizziness, and loss of consciousness (13). By this phenomenon, termed *hormesis*, CO may exert antiadipogenic, antilipogenic, anti-inflammatory. and/or antioxidant effects when administered at low, subtoxic doses.

Fibroblast growth factor 21 (FGF21) has emerged as a regulator of development, cell proliferation, and energy metabolism (17–20). FGF21 is an endocrine hormone that is produced predominantly in the liver but also in white adipose tissue (WAT), brown adipose tissue (BAT), pancreas, and skeletal muscle (21). Although FGF21 is expressed in multiple tissues, increases in the circulating levels of FGF21 have been linked to increased hepatic FGF21 synthesis (22). FGF21 is involved in the control of glucose homeostasis, insulin sensitivity, and ketogenesis (8, 20, 23, 24). Moreover, under cold conditions, FGF21 functions to increase the appearance of brown-like adipocytes in WAT depots (25). The expression of FGF21 is induced by peroxisome proliferator-activated receptors (PPARs)-α and -γ in various stresses, such as starvation, ketogenic diet, amino acid deprivation, high-fat diet (HFD) or obesity, low-carbohydrate levels, autophagy deficiency, and mitochondrial stress (26–28). The expression of FGF21 can also be regulated by ATF4, which is a downstream effector protein of the protein kinase R-like endoplasmic reticulum kinase (PERK) branch of the unfolded protein response (29, 30). Among the three known endoplasmic reticulum (ER) stress-sensing pathways: PERK, activating transcription factor 6 (ATF6), and inositol-requiring transmembrane kinase/endonuclease 1α (IRE1α), CO can selectively stimulate PERK activation through enhanced reactive oxygen species (ROS) production (31). PERK activation by CO increased elongation initiation factor 2α (eIF2α) phosphorylation and subsequently ATF4 expression (31). Here, we report that ATF4 expression elevated by CO resulted in increased FGF21 levels in hepatocytes and serum. FGF21 increased by CO stimulated the emergence of BATs from WATs. We also demonstrate that CO inhalation ameliorates HFD- and ER stress–induced hepatic steatosis and reduces insulin resistance and triglyceride levels. Therefore, we suggest that CO acts as a potent mediator of metabolic homeostasis by increasing endogenous FGF21 expression, which may have therapeutic implications in nonalcoholic steatohepatitis, obesity, type 2 diabetes, and related metabolic disorders.

## MATERIALS AND METHODS

### Reagents

Tunicamycin (Tm), MitoTEMPO, and CORM-2 were from MilliporeSigma (Billerica, MA, USA).

### Cell culture

Acute myeloid leukemia (AML)12 mouse hepatocytes (CRL-2254; American Type Culture Collection, Manassas, VA, USA) were cultured in DMEM/F12 (Thermo Fisher Scientific, Waltham, MA, USA) containing 10% fetal bovine serum and 1% penicillin-streptomycin solution at 37°C in a humidified incubator containing 5% CO_2_. Protein kinase R-like ER kinase deficient (*Perk*^−/−^) or wild-type (*Perk* ^+/+^) mouse embryonic fibroblasts (MEFs) were cultured in DMEM medium and 1% MEM nonessential amino acid solution (11140-050; Thermo Fisher Scientific). *Ire1α*^+/+^, *Ire1α*^−/−^, *Atf6α*^+/+^, *Atf6α*^−/−^, *Atf4*^+/+^, and *Atf4*^−/−^ mouse hepatocytes were maintained in 199 medium with 1% MEM nonessential amino acid solution and 2.2 μl β-mercaptoethanol.

### Isolation of primary hepatocytes

Livers were perfused with Ca^2+^- and Mg^2+^-free HBSS containing EGTA (2.5 mM) and then digested with a collagenase buffer containing collagenase (0.5 mg/ml, C5138; MilliporeSigma), NaCl (66.7 mM), KCl (6.7 mM), 4-(2-hydroxyethyl)-1-piperazineethanesulfonic acid (HEPES; 50 mM), and CaCl_2_ (4.8 mM). Digested livers were dissected and then gently teased with forceps until they were in solution. The cell suspensions were filtered through 100-μm nylon cell strainer (BD Biosciences, Franklin Lakes, NJ, USA). The cells were centrifuged for 3 min at 700 rpm and resuspended with HBSS. After the pellet suspensions were centrifuged with 25% Percoll for 5 min at 800 rpm with the brake option off, the pellets were washed with DMEM supplemented with 10% fetal bovine serum, and then, cells were seeded into collagen-precoated, 100-mm tissue-culture plates. After 24 h, nonadherent cells were removed by aspiration, and fresh medium was added.

### Animal experiments

All experiments with mice were approved by the Animal Care Committee of the University of Ulsan. C57BL/6 mice (6 wk old, male) were purchased from Orient Bio (Seongnam, Korea). The mice were maintained under specific pathogen-free conditions and given access to food and water *ad libitum*. To produce mice with diet-induced obesity, 6-wk-old *Fgf21*^+/+^ and *Fgf21*^−/−^ mice (6 mice/group) were fed an HFD (D12492; Research Diet, New Brunswick, NJ, USA) or normal chow diet (NCD) for 16 wk. Starting in wk 8, the mice were subjected to inhalation of CO gas [250 parts per million (ppm)] in air (Coregas, Yenora, NSW, Australia) for 2 h/d for the remaining 8 wk. To model ER stress–induced hepatosteatosis, 6-wk-old C57BL/6 mice were pretreated with or without CORM-2 (20 mg/kg) for 6 h, followed by stimulation with Tm (1 mg/kg). Tissue harvest was performed after 24 h. Heterozygous *eIF2α*^S/A^/*fTg* mice and *eIF2*^A/A^ mutant mice were kindly provided by Dr. S. H. Back (University of Ulsan).

### RT-PCR

Total RNA from cells was isolated with Trizol reagent (Thermo Fisher Scientific), according to the manufacturer’s instructions. In brief, total RNA (2 μg) was used to synthesize the first strand cDNA with oligo (dT) primer (Bioneer, Daejeon, Korea) and Moloney murine leukemia virus reverse transcriptase (Promega, Madson, WI, USA). The synthesized cDNA was subjected to the PCR-based amplification. The PCR products were detected on 2% agarose gels. The expression of glyceraldehyde-3-phosphate dehydrogenase (GAPDH) was measured as an internal control. The following primers were used: mGAPDH forward, 5′-AGGCCGGTGCTGAGTATGTC-3′, reverse, 5′-TGCCTGCTTCACCACCTTCT-3′; mFGF21 forward, 5′-ACAGATGACGACCAAGACACTG-3′, reverse, 5′-GTCCTCCAGCAGCAGTTCTC-3′; mPERK forward, 5′-ACTGTTGGCTGGCTCTCACT-3′, reverse, 5′-TGCCTTCGGGGTTAGTTATG-3′; mATF4, forward, 5′-GCAAGGATGCCTTTTC-3′, reverse, 5′-GTTTCCAGGTCATCCATTCG-3′; mNRF-1 forward, 5′-CTCCAAACCCAACCCTGTCT-3′, reverse, 5′-TGGTGGCCTGAGTTTGTGTT-3′; and mTFAM forward, 5′-CAGCCAGGTCCAGCTCACTA-3′, reverse, 5′-ATTAGGAGGGTCTCGCTCCA-3′.

### Real-time quantitative RT-PCR

Total RNA was prepared using Trizol reagent (Thermo Fisher Scientific); 2 µg of total RNA was used to synthesize the first-strand cDNA with oligo (dT) primers and Moloney murine leukemia virus reverse transcriptase (Promega), according to the manufacturer’s instructions. Real-time quantitative RT-PCR (qRT-PCR) was performed with SYBR Green qPCR MASTER Mix (2×, USB production; Thermo Fisher Scientific) on an ABI 7500 Fast Real-Time PCR System (Thermo Fisher Scientific). Real-time PCR primer pairs are listed in Supplemental Table 1.

### Western blot analysis

Harvested liver tissues and cells were lysed in RIPA buffer containing phosphatase and protease inhibitors. Equal amounts of cell lysates was measured by BCA protein assay reagent (Thermo Fisher Scientific). Lysates were boiled in sample buffer containing β-mercaptoethanol for 5 min. Proteins were then subjected to SDS-PAGE and transferred to PVDF membranes (SigmaMillipore). After blocking with 5% skim milk in PBS, membranes were incubated with appropriate dilutions of antibodies at 4°C overnight as follows: anti–α-tubulin (Cell Signaling Technology, Danvers, MA, USA), anti–phospho-PERK (Cell Signaling Technology), anti-PERK (Cell Signaling Technology), anti–phospho-eIF2α (Cell Signaling Technology), anti-eIF2α (Cell Signaling Technology), anti-ATF4 (Santa Cruz Biotechnology, Santa Cruz, CA, USA), anti–phospho-AMPK (Cell Signaling Technology), anti-AMPK (Cell Signaling Technology), and anti-PGC1α (Abcam, Milton, Cambridge, United Kingdom), anti-COX III (Abcam), and anti-COX IV (Cell Signaling Technology). Membranes were then washed with 0.05% PBS-Tween 20 and incubated with 1/5000 dilution of horseradish peroxidase–conjugated secondary antibodies at room temperature for 1 h. Immunoreactivity was detected with an enhanced chemilumescent detection system (GE Healthcare Life Sciences, Little Chalfont St Giles, United Kingdom). Chemiluminescence signals were read with an Azure Biosystems C300 analyzer (Azure Biosystems, Dublin, CA, USA).

### Hematoxylin and eosin staining

To detect the pathologic changes, portions of the liver were fixed in 10% neutral-buffered formalin solution and then dehydrated in graded alcohol, embedded in paraffin, and sectioned into 4-μm–thick sections. Tissue sections were mounted on regular glass slides, deparaffinized in xylene, rehydrated in decreasing concentrations of ethanol, and stained with hematoxylin and eosin (H&E).

### Small interfering RNA transfection

Small interfering RNAs (siRNAs) against mouse *Fgf*21 (sc-39485) and *Perk* (sc-36214) were purchased from Santa Cruz Biotechnology, and negative control siRNA (scRNA; AM4611) was purchased from Ambion (Austin, TX, USA). AML12 cells (7 × 10^5^) were transfected with siRNAs with Lipofectamine 2000 (Thermo Fisher Scientific) for 24 h.

### Hepatocellular damage assay

Serum alanine aminotransferase (ALT) and aspartate aminotransferase (AST) activity, as indicators of hepatocellular injury, were measured with the EnzyChrom Alanine Transaminase assay kit and the EnzyChrom Aspartate Transaminase Assay Kit (Bio Assay Systems, Hayward, CA, USA).

### Measurement of triglycerides

Hepatic triglycerides (TGs) were assessed with a TG colorimetric assay kit (Cayman Chemicals, Ann Arbor, MI, USA). Briefly, liver tissues (50 mg) were homogenized in 200 μl diluted standard diluents. After centrifugation for 10 min at 10,000 *g*, supernatants were obtained. Before assaying, tissue samples required dilutions of ≥1:5, and 10 μl supernatant was used for the assay.

### Oxygen consumption rate measurement

Oxygen consumption rate (OCR) was measured with an XF24 Extracellular Flux Analyzer (Seahorse Bioscience, North Billerica, MA, USA), based on the fluorometric detection of O_2_ and pH levels. The day before the assay, the cells were seeded in XF24 microplates at 10,000 cells/well in 250 μl of complete culture medium and were maintained at 37°C in a humidified incubator containing 5% CO_2_; the cells were fully confluent on the day of the assay. The culture medium in the XF24 microplate was replaced with 525 µl/well of standard assay medium (Seahorse Bioscience). The cells were equilibrated in that assay medium for 1 h at 37°C without CO_2_. The Seahorse cartridge was incubated in the calibration solution (Seahorse Bioscience) overnight before loading oligomycin (75 µl) in port A, FCCP (75 µl) in port B, and a combination of rotenone and antimycin A (75 µl) in port C (XF Cell Mito Stress Test Kit; Seahorse Bioscience). Following the calibration and equilibration of the loaded cartridge, OCR was periodically measured with a sequence of 3-min mix, 2-min wait, and 3-min measurement. Baseline rates were measured, as well as rates after addition of oligomycin, FCCP, and the combination of rotenone and antimycin A. The averages of the 3 measurements of each experiment were used for data analyses (the first reading of baseline was dropped for analysis). OCR is reported as picomoles per minute or percentage relative to the basal level of the individual experiment. All experiments were performed in at least hexaplicates.

### Glucose tolerance test and insulin tolerance test

Glucose tolerance tests (GTTs) were performed in overnight-unfed mice with an intraperitoneal injection of glucose (1 g/kg). An insulin tolerance test (ITT) was conducted in 4-h–unfed mice with an intraperitoneal injection of insulin (1 U/kg). Blood glucose levels were measure with SD Codefree blood glucose meter (SD Biosensor, Suwon, South Korea).

### Mitochondrial ROS measurement

AML12 cells were treated with CORM2 (20 μM) or CO gas (250 ppm) for 2 h in the absence or presence of Mito-Tempo (100 μM) or *N*-acetyl-l-cysteine (NAC; 3 mM). After treatment, cells were incubated in fresh medium and were treated with 5 μM MitoSox Red (Thermo Fisher Scientific) for 50 min at 37°C under dark conditions. Subsequently, cells were trypsinized, placed in fluorescence-activated cell sorting tubes, and washed 2 times with 1× PBS (Thermo Fisher Scientific). Samples were diluted to a final volume of 500 μl with 1× PBS. MitoSox fluorescence was detected by FACSCanto flow cytometry system (BD Biosciences). Data were analyzed with FlowJo software (v.10; Tree Star, Ashland, OR, USA).

### Statistical analysis

All data were expressed as means ± sem. Differences among experimental groups were compared using 1- or 2-way ANOVA analysis.

## RESULTS

### FGF21 is required for the protective effects of CO on HFD-induced metabolic syndrome in mice

**Figure 1.**
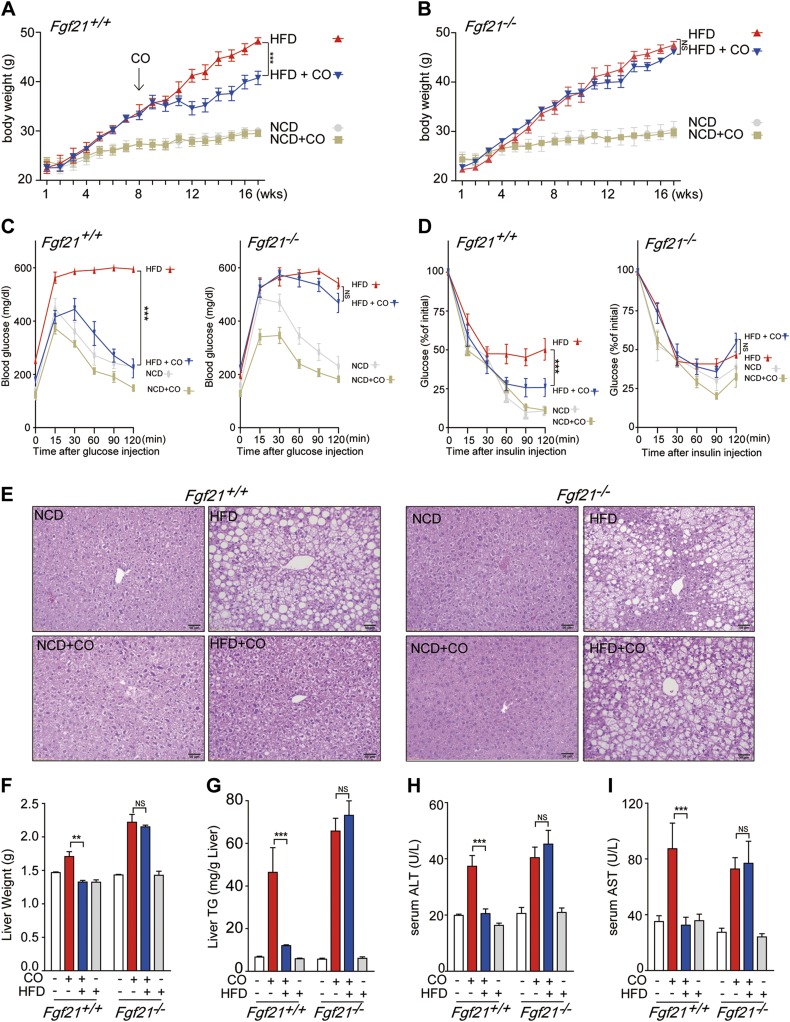
The protective effects of CO on HFD-induced metabolic syndrome are mediated by FGF21. *A*, *B*) Male, 6-wk-old C57BL/6 mice (6 mice/group) were fed an NCD or HFD for 16 wk. After 8 wk, animals were subjected to inhaled CO (250 ppm) for 2 h/d for 8 wk. The average body weight of *Fgf21^+/+^* (*A*) and *Fgf21^−/−^* (*B*) mice was measured every 2 d. *C*, *D*) GTT (*C*) and ITT (*D*) were determined after 16 wk of NCD and HFD feeding. *E*) H&E staining of liver tissues was performed in *Fgf21^+/+^* and *Fgf21^−/−^* mice. Scale bars, 50 μm. *F*–*I*) Liver weight (*F*), triglyceride (*G*), serum ALT (*H*), and AST (*I*) levels were investigated after 16 wk of NCD and HFD feeding. Data are presented as means ± sem (*n* = 6). NS, not significant. **P < 0.01, ***P < 0.001.

FGF21, which is expressed in multiple tissues, is a regulator of glucose homeostasis and insulin sensitivity. We first examined whether FGF21 is involved in the protective effects of CO against obesity-mediated metabolic syndrome. Wild-type (*Fgf21*^+/+^) and mice genetically deficient in FGF21 (*Fgf21*^−/−^) were maintained on an HFD or NCD and then subjected to CO inhalation (250 ppm, beginning at 8 wk). As shown in **Fig. 1*A***, the body weight of wild-type *Fgf21*^+/+^ mice on HFD was dramatically decreased by CO inhalation. In contrast, CO inhalation did not alter body weight in *Fgf21*^−/−^ mice, despite lack of significant difference in food intake between the strains (Fig. 1*B*). In NCD-fed mice, there was no measurable difference in body weight after CO inhalation in either strain. There was no significant difference in body weight observed between *Fgf21*^+/+^ mice and *Fgf21*^−/−^ mice fed with either NCD or HFD (Supplemental Fig. 1). CO inhalation resulted in significant improvement in glucose tolerance (Fig. 1*C*). CO inhalation did not improve glucose tolerance in HFD-fed *Fgf21*^−/−^ mice. CO inhalation also improved insulin sensitivity as assessed by an insulin tolerance test in HFD-fed *Fgf21*^+/+^ mice (Fig. 1*D*). Similar to glucose tolerance, CO inhalation did not affect insulin sensitivity in HFD-fed *Fgf21*^−/−^ mice. As expected, neither glucose tolerance nor insulin sensitivity was significantly altered by CO inhalation in either *Fgf21*^−/−^ or *Fgf21*^+/+^ mice on NCD. In addition, CO inhalation improved hepatocyte derangement and excessive vacuole formation in the livers of HFD-fed *Fgf21*^+/+^ mice but not *Fgf21*^−/−^ mice (Fig. 1*E*). Moreover, liver weight and liver TGs, which were increased by HFD were decreased by CO inhalation in *Fgf21*^+/+^ mice, but not *Fgf21*^−/−^ mice (Fig. 1*F*, *G*). We next demonstrated that the liver injury markers ALT and AST were dramatically reduced by CO inhalation in HFD-fed *Fgf21*^+/+^ mice, when compared with those observed in HFD-fed *Fgf21*^−/−^ mice (Fig. 1*H*, *I*). These data indicate that FGF21 is essential for the protective effects of CO in metabolic syndrome.

### FGF21 expression is increased by CO through increased ROS production

**Figure 2.**
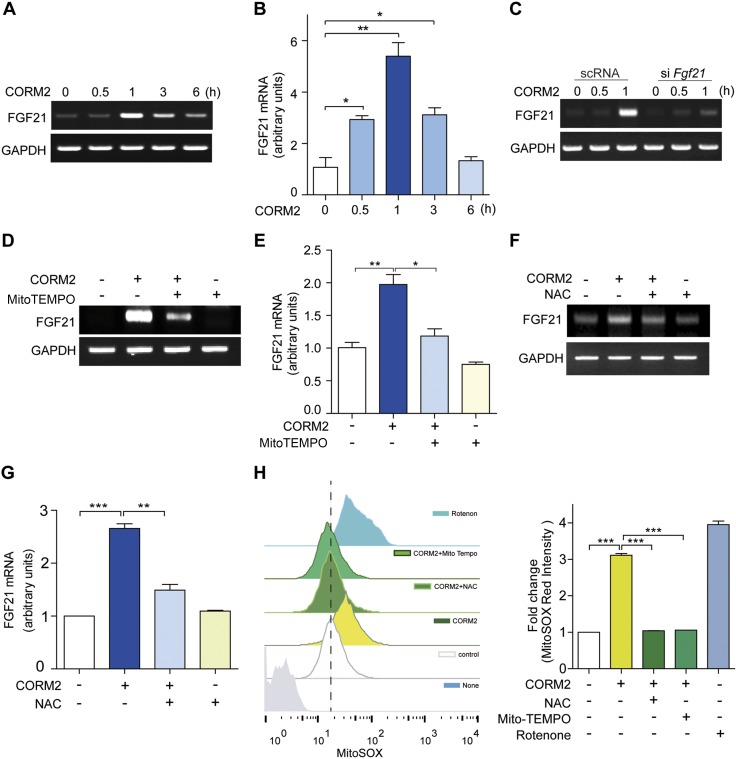
CO enhances the expression of FGF21 *via* ROS production. *A*, *B*) AML12 cells were treated with CORM2 (20 μM) for various times (0, 0.5, 1, 3, and 6 h), and then, the FGF21 mRNA levels were measured by RT-PCR (*A*) and qRT-PCR (*B*). *C*) AML12 cells were transfected with scRNA and siRNA against *Fgf21*, followed by the administration of CORM2 (20 μM) for 0.5 and 1 h. *D*, *E*) AML12 cells were pretreated with MitoTEMPO (100 μM) for 1 h, and then, cells were treated with CORM2 (20 μM) for another 2 h. Then, the mRNA expression of FGF21 was determined by RT-PCR (*D*) and qRT-PCR (*E*). *F*, *G*) AML12 cells were pretreated with NAC (3 mM) for 30 min, followed by the administration of CORM2 (20 μM) for another 2 h. Then, the mRNA expression of FGF21 was detected by RT-PCR (*F*) and qRT-PCR (*G*). *H*) To assess the production of mtROS, AML12 cells were pretreated with NAC (3 mM) for 30 min and MitoTEMPO (100 μM) for 1 h, and then were treated with CORM2 (20 μM) for another 2 h. mtROS was detected with MitoSOX Red (5 μM) for 50 min and measured by flow cytometry. Rotenone (10 μM) was used as a positive control for mtROS production. Data are presented as means ± sem (*n* = 3). NS, not significant. *P < 0.05, **P < 0.01, ***P < 0.001.

We next investigated the mechanisms by which CO can increase FGF21 expression. Application of CORM2 or exposure to CO gas increased FGF21 expression in AML12 cells or primary hepatocytes (**Fig. 2*A*, *B*** and Supplemental Fig. 2A, B). CORM2 (20 μM, 1 h treatment) stimulated the increase of FGF21 mRNA expression in AML12 cells (Fig. 2*A*), which was confirmed by qRT-PCR analysis (Fig. 2*B*). CO exposure (0–5 h, 250 ppm) increased FGF21 expression at 5 h in primary hepatocytes (Supplemental Fig. 2*A*). Increased *FGF21* gene expression was confirmed by qRT-PCR analysis (Supplemental Fig. 2*B*). Knockdown of FGF21 using siRNA against *Fgf21* (si*Fgf21*), prevented up-regulation of FGF21 expression by CORM2 treatment, compared with control siRNA transfected cells (Fig. 2*C*). These results indicate that FGF21 expression is transcriptionally increased by CO. We also determined whether the increase of FGF21 by CORM2 or CO gas involved mitochondrial ROS (mtROS) generation, using the specific mitochondria-targeted, antioxidant mitoTEMPO (Fig. 2*D*, *E* and Supplemental Fig. 2*C*, *D*). The increase of FGF21 expression with CORM2 was reduced by mitoTEMPO. We also treated CORM2 or CO-stimulated AML cells with NAC, a general ROS inhibitor. As shown in Fig. 2*F*, *G* and Supplemental Fig. 2*E*, *F* the increase of FGF21 expression by CORM2 or CO was inhibited by NAC treatment. To determine whether the administration of CORM2 or CO gas could affect mtROS production, we next performed flow cytometry using MitoSox. As expected, treatment with CORM2 (Fig. 2*H*) or CO gas (Supplemental Fig. 2*G*) increased mtROS at 2 h in AML12 cells. Moreover, pretreatment with NAC or MitoTEMPO significantly decreased ROS production in the presence of CORM2 (Fig. 2*H*) or CO gas (Supplemental Fig. 2*G*). The mitochondrial complex I inhibitor rotenone (10 μM) was used as a positive control for mtROS production. Those results suggested that ROS production serves an important role in up-regulating FGF21 expression by CO.

### PERK activation by CO mediates the regulation of FGF21 expression

**Figure 3.**
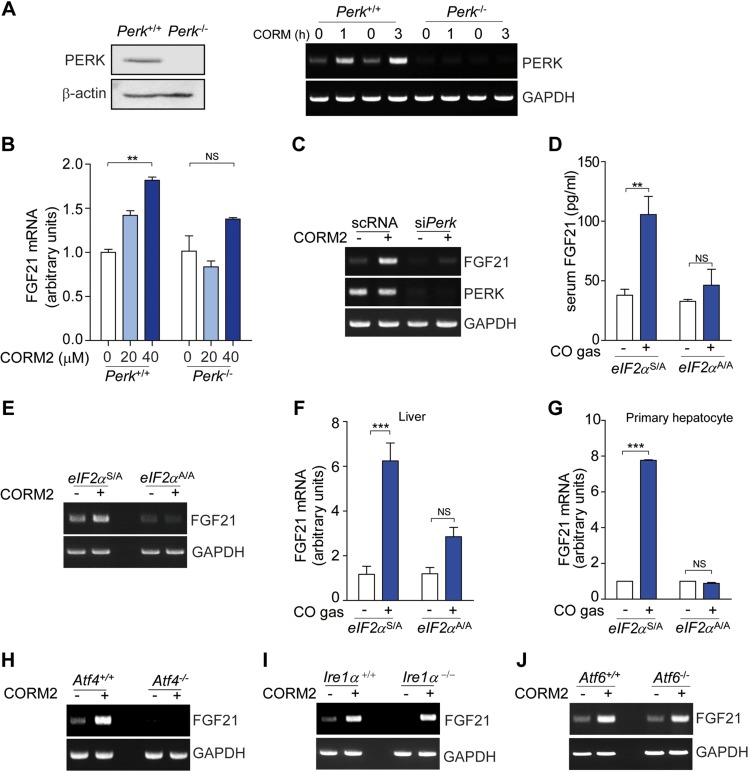
The expression of FGF21 regulated by CO is mediated by PERK signaling pathway. *A*, *B*) MEF cells from *Perk^+/+^* and *Perk^−/−^* mice were treated with CORM2 (20 μM) for 1 and 3 h. Then, FGF21 mRNA expression was determined by RT-PCR (*A*). *Perk^+/+^* and *Perk^−/−^* MEF cells were treated with CORM2 at various concentrations (0, 20, and 40 μM) for 3 h, and the mRNA level of FGF21 was measured by qRT-PCR (*B*). *C*) AML12 cells were transfected with scRNA and siRNA against *Perk*, and then, cells were treated with CORM2 (20 μM) for 3 h. mRNA levels of FGF21 and PERK were detected by RT-PCR. *D*–*F*) Heterozygous *eIF2α*^S/A^/*fTg* mice and *eIF2*^A/A^ mutant mice were administrated CORM-2 for 3 h or CO gas (250 ppm) for 2 h. Serum FGF21 levels were assessed by ELISA (*D*), and mRNA levels in liver tissues were assessed by RT-PCR (*E*) and qRT-PCR (*F*). *G*) Primary hepatocytes isolated from heterozygous *eIF2α*^S/A^/*fTg* mice and *eIF2*^A/A^ mutant mice were treated with CO gas for 2 h, and the mRNA level of FGF21 was detected by qRT-PCR. *H*–*J*) Hepatocytes from *Atf4^+/+^*, *Atf4^−/−^*, *Ire1^+/+^*, *Ire1^−/−^*, *Atf6^+/+^*, and *Atf6^−/−^* mice were treated with CORM2 or RuCl_2_ as a control for ruthenium for 3 h to assess the mRNA expression of FGF21 by RT-PCR. Data are presented as means ± sem (*n* = 3). NS, not significant. **P < 0.01, ***P < 0.001.

We next explored the molecular mechanisms by which FGF21 expression was regulated by the PERK pathway. In our previous study (31), we reported that CO activated PERK but not other eIF2α kinase family proteins, such as hemin-regulated inhibitor kinase, general control of amino acid biosynthesis kinase, or protein kinase R. We also demonstrated that CO induced ATF4 expression *via* the PERK-mediated eIF2α pathway (31). Based on our studies, we assesed whether FGF21 expression was dependent on PERK activation. As shown in **Fig. 3*A***, *FGF21* gene expression was increased by CORM2 treatment in MEF cells isolated from *Perk*^+/+^ mice, but not *Perk*^−/−^ mice. These results were also confirmed by qRT-PCR analysis (Fig. 3*B*). Furthermore, knockdown of PERK using siRNA also prevented the increase of *FGF21* gene expression in response to CORM2 (Fig. 3*C*). PERK activation by CO is associated by eIF2α phophorylation and increases in ATF4 expression (31). To further identify the mechanism by which CO-induced activation of the PERK pathway stimulates FGF21 expression and the involvement of eIF2α and ATF4, we first compared the levels of FGF21 expression between control heterozygous *eIF2α*^S/A^/*fTg* mice and *eIF2α*^A/A^ mutant mice after CO inhalation. The levels of FGF21 in serum were increased by CO inhalation in control heterozygous *eIF2α*^S/A^/*fTg* mice, but not in *eIF2α*^A/A^ mutant mice (Fig. 3*D*). Next, we assessed FGF21 expression in the livers or in primary hepatocytes from control heterozygous *eIF2α*^S/A^/*fTg* mice and *eIF2α*^A/A^ mutant mice. The mRNA expression of *Fgf21* in response to CORM2 treatment was greatly elevated in the liver tissues of control heterozygous *eIF2α*^S/A^/*fTg* mice, compared with those of *eIF2α*^A/A^ mutant mice (Fig. 3*E*). The increase of FGF21 by CO inhalation in liver tissue from control heterozygous *eIF2α*^S/A^/*fTg* mice was investigated by qRT-PCR (Fig. 3*F*). In additon, FGF21 expression was markedly up-regulated by CO treatment in primary hepatocytes isolated from control heterozygous *eIF2α*^S/A^/*fTg* mice, compared with those of *eIF2α*^A/A^ mutant mice (Fig. 3*G*). ATF4 expression is mediated by the PERK-mediated eIF2α pathway (32) and regulates FGF21 expression (33, 34). To verify whether the increase of FGF21 expression by CO was also dependent on ATF4 expression, we assessed the levels of FGF21 expression in hepatocytes isolated from *Atf4^+/+^* mice and *Atf4^−/−^* mice after CORM2 treatment. The expression of FGF21 in response to CORM2 was greatly increased in hepatocytes from *Atf4^+/+^* mice, compared with *Atf4^−/−^* mice (Fig. 3*H*). We next investigated whether the other two known ER stress–sensing pathways, namely the IRE1α and the ATF6 pathways, could regulate the expression of FGF21 in response to CO, using hepatocytes isolated from *Ire1α^+/+^*, *Ire1α^−/−^*, *Atf6^+/+^*, and *Atf6^−/−^* mice. The ability of CO to induce the expression of *Fgf21* mRNA was not affected by genetic deficiency of IRE1α and ATF6 (Fig. 3*I*, *J*). Thus, CO increases FGF21 expression through the PERK-eIF2α-AFT4 pathway.

### Induction of FGF21 expression by CO ameliorates ER stress-induced hepatic steatosis

**Figure 4.**
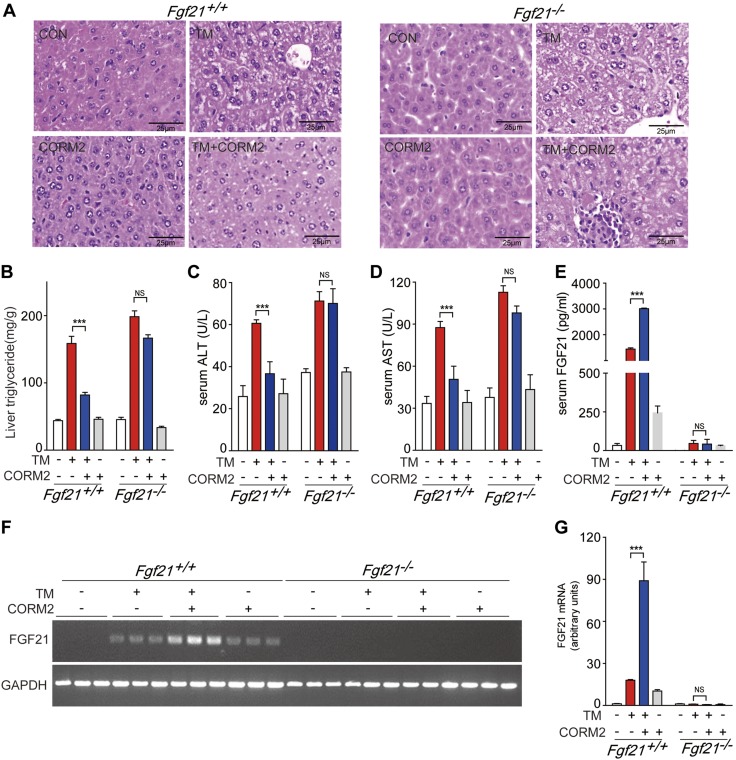
CO protects against ER stress-induced hepatic steatosis by induction of FGF21 expression. *A*) 6-wk-old *Fgf21^+/+^* and *Fgf21^−/−^* mice were pretreated with or without CORM2 (20 mg/kg) or RuCl_2_ as control for ruthenium, for 6 h, followed by Tm (1 mg/kg) challenge. After 24 h, the mice were euthanized, and liver tissues were extracted and analyzed by H&E staining. *B–D*) Liver triglyceride (*B*), serum ALT (*C*), and AST (*D*) levels were measured after stimulation with Tm. *E*) Serum FGF21 levels were detected by ELISA. *F*, *G*) The mRNA expression of FGF21 in liver tissues was measured by RT-PCR (*F*) and qRT-PCR (*G*). Data are presented as means ± sem (*n* = 3). NS, not significant. ***P < 0.001.

Obesity induces ER stress which in turn can promote hepatic steatosis (35). We previously reported that CO ameliorates ER stress-induced hepatic steatosis (36, 37). Thus, we investigated whether CO-induced FGF21 expression alleviates hepatic steatosis in *Fgf21^+/+^* and *Fgf21^−/−^* mice challenged with ER stressors. As shown in **Fig. 4*A***, administration of Tm, an inhibitor of *N*-linked glycosylation that causes accumulation of misfolded protein in the ER, induced hepatic injury in *Fgf21^+/+^* mice. CORM2 treatment protected against Tm-induced hepatic injury in *Fgf21^+/+^* mice but was ineffective in *Fgf21*^−/−^ mice. Furthermore, liver TG, ALT, and AST levels were increased by Tm in both *Fgf21^+/+^* and *Fgf21^−/−^* mice (Fig. 4*B–D*). CORM2 treatment reduced the levels of liver TG, AST, and ALT in *Fgf21^+/+^*, but not in *Fgf21^−/−^*, mice.

The expression of FGF21 is induced by ER stress through the PERK-eIF2α-ATF4 pathway (30). Tm treatment induced FGF21 in the serum of *Fgf21^+/+^* mice as an adaptation to ER stress. The addition of CORM2 further increased the FGF21 levels >2.5 times relative to Tm treatment alone (Fig. 4*E*). FGF21 levels were accordingly not increased in *Fgf21^−/−^* mice by either treatment. To investigate the mRNA expression of *Fgf21* in the liver after Tm and CORM2 treatment in *Fgf21^+/+^* and *Fgf21^−/−^* mice, we performed RT-PCR (Fig. 4*F*) and qRT-PCR (Fig. 4*G*). CORM2 greatly elevated the expression of FGF21 mRNA in the Tm- and CORM2-treated group. CORM2 promoted recovery from ER stress by alleviating proinflammatory cytokine production (*i.e.*, IL-6, IL-1β, and TNF-α), and that was dependent on the presence of FGF21 (Supplemental Fig. 3*A*). In addition, we found that Tm-induced hepatocyte damage in the liver was alleviated by administration of rmFGF21 (Supplemental Fig. 3*B*). Furthermore, the levels of ALT and liver TGs increased in response to Tm were reduced by application of exogenous FGF21 (Supplemental Fig. 3*C*, *D*). These data indicate that exogenous FGF21 reduces liver damage caused by Tm and thus has an important role in the regulation of ER stress-induced hepatic steatosis.

### CO regulates lipid metabolism and energy expenditure through the induction of FGF21 expression

**Figure 5.**
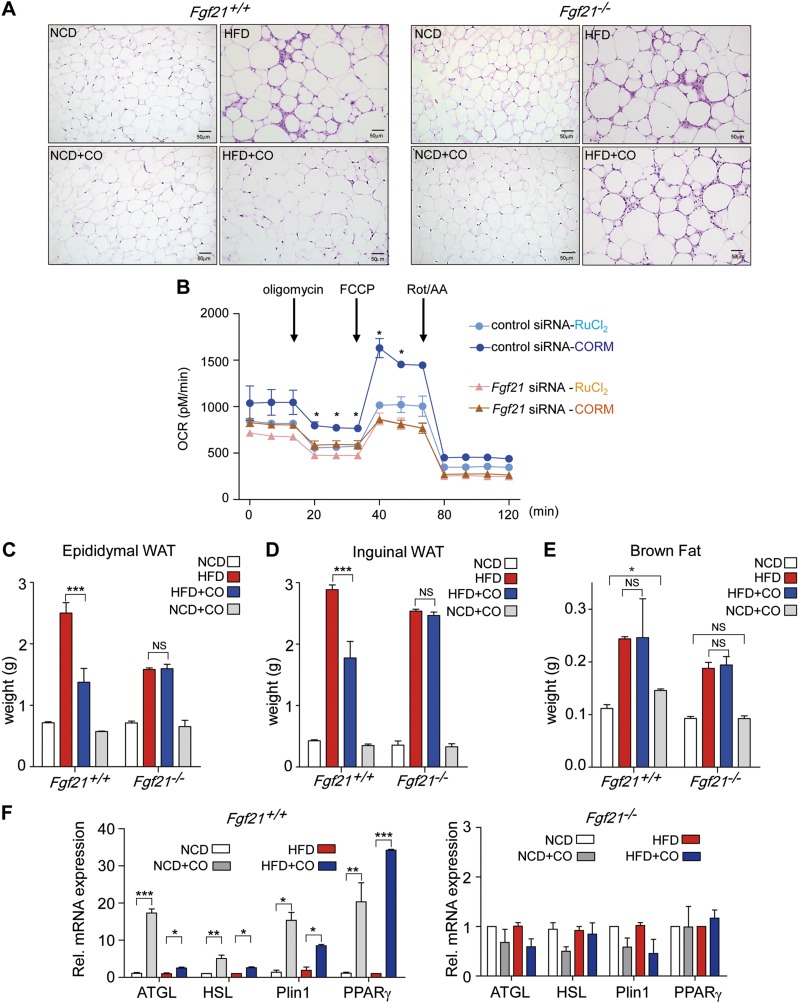
CO regulates lipolysis through the induction of FGF21 expression. *A*) H&E staining of epididymal WAT from *Fgf21^+/+^* and *Fgf21^−/−^* mice that had been fed a NCD or HFD for 16 wk with CO inhalation. *B*) OCR was measured in AML12 cells transfected with scRNA and siRNA against *Fgf21* in the presence of CORM2 (20 μM) and RuCl_2_ as control for ruthenium to detect energy expenditure and mitochondrial function. *C*–*E*) The weight of eWAT (*C*), iWAT (*D*), and brown fat (*E*) was investigated after 16 wk of NCD and HFD feeding with CO inhalation. *F*) The lipolysis-related genes (*ATGL*, *HSL*, *Plin1*, and *PPARγ*) from *Fgf21^+/+^* and *Fgf21^−/−^* eWAT were measured by qRT-PCR. Data are presented as means ± sem (*n* = 6). NS, not significant. *P < 0.05; **P < 0.01, ***P < 0.001.

To assess directly whether induction of FGF21 expression by CO inhalation is responsible for a protective role against metabolic dysfunction, we investigated metabolic changes in *Fgf21^+/+^* or *Fgf21^−/−^* mice fed with HFD. Histologic analysis revealed that the size of adipocytes were increased by HFD and were decreased by CO treatment in *Fgf21^+/+^* mice but not in *Fgf21^−/−^* mice (**Fig. 5*A***). We also found that CORM2 treatment increased the OCR in the AML12 mouse hepatocyte cell line, relative to the control groups (Fig. 5*B*). Genetic interference of FGF21 prevented the increase of OCR elicited by CORM2 treatment in those cells. The measurement of OCR may represent an increase in energy expenditure or mitochondrial function. To further study the mechanisms by which CO can alter mitochondrial function or mitochondrial biogenesis, we examined the effect of CORM2 on the expression of components of mitochondrial respiratory chain subunits (COX III, COX IV), and cellular mtDNA levels using wild-type or FGF21-deficient AML2 cells (Supplemental Fig. 4*A–D*) and primary hepatocytes from *Fgf21^−/−^* or *Fgf21^+/+^* mice (Supplemental Fig. 4E–H). CORM2 treatment resulted in increased expression of COX III and COX IV protein and increased mtDNA levels in control (scRNA) AML12 cells (Supplemental Fig. 4*B*, *C*) and wild-type primary hepatocytes (Supplemental Fig. 4*F*, *G*). In AML2 cells transfected with siFGF21, knockdown of FGF21 was validated in Supplemental Fig. 4*A*. In FGF21 knockdown cells, CORM2 treatment failed to increase COX III and COX IV protein expression and mtDNA levels, relative to CORM2-treated control (sc-RNA transfected) cells (Supplemental Fig. 4*B*, *C*). Furthermore, in primary hepatocytes deficient in FGF21 (*Fgf21*^−/−^) (Supplemental Fig. 4*E*), CORM2 treatment failed to increase COX III and COX IV protein expression and mtDNA levels, relative to CORM2-treated wild-type (*Fgf21*^+/+^) hepatocytes (Supplemental Fig. 4*F*, *G*).

We also investigated the effects of CORM2 on mitochondrial functions by measuring the expression levels of peroxisome proliferator-activated receptor γ coactivator 1-α (PGC1α), mitochondrial transcription factor-A (TFAM), and nuclear respiratory factor-1 (NRF-1), protein factors critical in the regulation of mitochondrial biogenesis. As shown in Supplemental Fig. 4D, H, CORM2 increased the PGC1α, TFAM, and NRF-1 mRNA levels in AML12 cells transfected with scRNA and in primary hepatocytes from *Fgf21^+/+^* mice but not in corresponding FGF21-deficient (siFGF21-transfected or *Fgf21*^−/−^) control hepatocytes. These data suggest that CORM2 regulates mitochondrial biogenesis and function *via* FGF21 increase. These findings indicate that CO-induced FGF21 expression has an important role in energy balance in HFD-induced metabolic syndrome, *via* regulating mitochondrial functions.

We also investigated whether the regulation of energy balance by CO-induced FGF21 involved alteration of fatty acid metabolism. We first measured the various adipose tissue weights after CO inhalation in *Fgf21^+/+^* or *Fgf21^−/−^* mice fed with HFD. As shown in Fig. 5*C–E*, the weight of epididymal WAT (eWAT) or inguinal WAT (iWAT) was increased with HFD, and was decreased by CO in *Fgf21^+/+^* mice, but not in *Fgf21^−/−^* mice (Fig. 5*C*, *D*). CO also increased BAT weight in NCD-fed wild-type (*Fgf21^+/+^*) mice. The BAT weight was unchanged in NCD-fed *Fgf21^−/−^* mice or in either *Fgf21^+/+^* or *Fgf21^−/−^* mice fed an HFD (Fig. 5*E*). Gene expression analysis revealed that CO inhalation elicited a marked increase in mRNA levels of lipolytic enzymes in eWAT, including adipose triglyceride lipase (ATGL), hormone-sensitive lipase (HSL), perilipin 1A (Plin1), and PPARγ in either NCD- or HFD-fed *Fgf21^+/+^* mice, but not in*Fgf21^−/−^* mice (Fig. 5*F*). To investigate liver lipolysis, we measured the mRNA expression of PGC1α, ATGL, HSL, lipolysis-stimulated lipoprotein receptor, Plin1, CPT1b, and PPARα in liver tissues (Supplemental Fig. 5). CO increased the mRNA expression levels of lipolytic enzymes in *Fgf21^+/+^* mice regardless of diet (NCD or HFD fed) but did not have an effect in *Fgf21^−/−^* mice. We could not detect any significant difference in lipogenic enzyme gene expression in response to CO inhalation in either *Fgf21^+/+^* mice or *Fgf21^−/−^* mice (data not shown). These findings demonstrate that CO regulates lipolysis in the WAT and liver in an FGF21-dependent manner.

### CO-mediated induction of thermogenic genes is regulated by FGF21 expression

**Figure 6.**
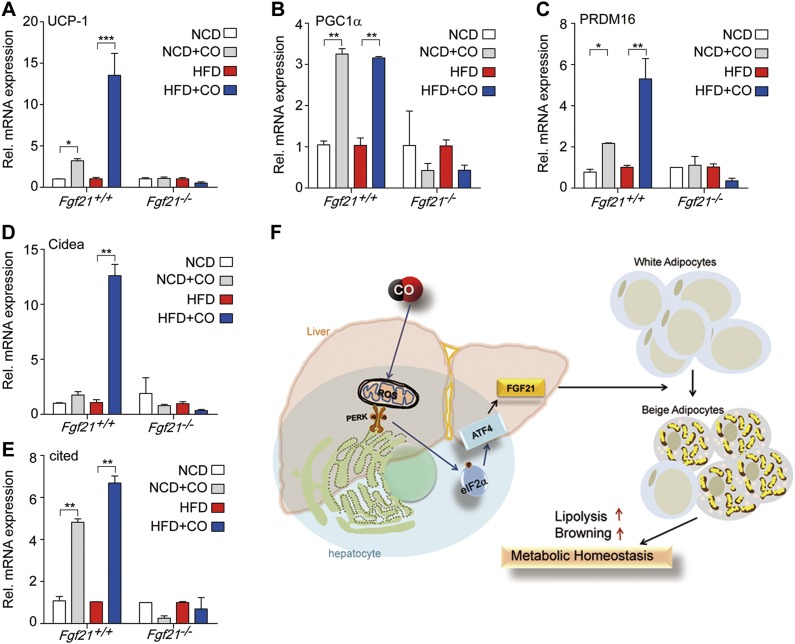
CO-mediated induction of thermogenic genes is regulated by the FGF21 expression. *A*–*E*) The mRNA expression of genes [*UCP1* (*A*), *PGC1α* (*B*), *PRDM16* (*C*), *Cidea* (*D*), and *Cited* (*E*)] associated with thermogenesis from *Fgf21^+/+^* and *Fgf21^−/−^* iWAT, were detected by qRT-PCR. Data are presented as means ± sem (*n* = 6). NS, not significant. *P < 0.05, **P < 0.01, ***P < 0.001. *F*) In mice with diet-induced obesity, CO increases FGF21 expression mediated by the PERK-ATF4 signaling pathway, which induces lipolysis and thermogenesis of WAT to regulate the metabolic homeostasis.

The regulation of metabolic homeostasis is associated with white adipocyte metabolism as well as the browning of white adipose depots. FGF21 is a critical regulator of the thermogenic activity of adipose tissue. Therefore, we investigated whether the induction of thermogenic genes by CO required FGF21 expression. CO inhalation resulted in a dramatic increase in the expression of genes normally associated with the browning of iWAT, such as uncoupling protein-1 (UCP1) (**Fig. 6*A***), PGC1α (Fig. 6*B*), PRDM16 (Fig. 6*C*), Cidea (Fig. 6*D*), and Cited (Fig. 6*E*) in *Fgf21^+/+^* mice. In contrast, there were no significant changes in the expression of those genes in iWAT of *Fgf21^−/−^* mice. Therefore, we conclude that the increase in the browning of iWAT by CO is dependent on FGF21 expression. We present a scheme in Fig. 6*F* for the role of CO in the control of metabolic homeostasis. CO increases PERK activation *via* mtROS production. Subsequently, FGF21 expression induced by CO *via* the PERK-ATF4 pathway regulates lipolysis and thermogenesis.

## DISCUSSION

In the current study, we demonstrated that CO induces FGF21 expression, which, along with previous studies (12), provides a mechanism by which CO regulates metabolic homeostasis. Although CO can be poisonous when inhaled in air at excess of 1500 ppm (13), CO can exert various therapeutic effects in preclinical models of lung injury and disease, either as an endogenous mediator or in response to exogenous inhalation or pharmacologic delivery (38, 39). Here, we demonstrate that CO-dependent hormesis includes an antiobesity effect at a low dose (250 ppm) *via* increasing FGF21 expression. Moreover, we show that CO induces FGF21 expression in primary hepatocytes as well as in metabolic tissues, such as liver and WAT.

The increase of FGF21 expression by CO is associated with alleviating metabolic disorders, including improving glucose tolerance and insulin sensitivity and in ameliorating hepatic steatosis and adipose tissue dysfunction. Notably, increased FGF21 expression was required for the effects of CO on metabolic disease during HFD feeding *in vivo*. FGF21 expression is induced by ER stress *via* ATF4 overexpression and ATF4-induced promoter activity (40), activation of CHOP-dependent (34) and IRE1α-XBP1 pathways (41) and stimulation of the PERK-eIF2α-ATF4 axis (30). In this study, we demonstrated that the mechanism by which CO-induced FGF21 expression primarily involves stimulation of the PERK-eIF2α-ATF4 axis. CO, however, failed to induce the IRE1α-mediated Xbp1 pathway or protease-mediated ATF-6 cleavage, as shown in our previous report (31). Our present results are consistent with selective PERK activation by CO, which excludes the IRE1α and ATF6 pathways. We also demonstrated here that PERK activation by CO requires up-regulation of mtROS production. Both NAC and the mitochondria-targeting antioxidant MitoTEMPO inhibited CO-induced FGF21 expression. Another report (16) has shown that mitochondria-derived ROS were required for CO-dependent induction of autophagy. We also demonstrate that CO up-regulated other mitochondrial functions, including the expression of electron transport chain complexes (COX III, COX IV) and mitochondrial biogenesis (as evidenced by increased mtDNA copy number and increased gene expression of mitochondrial biogenesis factors) and that those effects were diminished in hepatocytes genetically deficient in FGF21. The mechanism for FGF21 to enhance mitochondrial function remains unclear. However, there are some reports that FGF21 enhances mitochondrial functions in human dopaminergic neurons (*via* the Sirt1-dependent regulation of PGC-1α) (42) and in skeletal muscle cells (43).

These results, taken together, suggested that CO targets mitochondrial functions, in a fashion that requires the presence of FGF21. Additionally, a recent article reported that FGF21 expression is increased by application of an ER stressor compound in hepatocytes (40). FGF21 is induced as a compensatory mechanism by various stresses, such as glucose starvation, cold shock, and autophagy deficiency (25, 44, 45). ER stress exerts an important pathogenic mechanism in various metabolic disorders, including the development of nonalcoholic fatty liver disease (35, 46, 47). Recombinant FGF21 protein could ameliorate hepatic liver injury that was caused by typical ER stress (41). Therefore, the reduction of ER stress by CO may result from FGF21-induced suppression of lipid accumulation and inflammation. In other reports, FGF21 was shown to prevent increased hepatic fat accumulation and enhanced hepatic inflammation in response to alcohol, endotoxin, or HFD (48–50). Our study indicated that CO-induced FGF21 expression attenuates liver damage and hepatic lipid accumulation resulting from ER stress. We also showed that CO can ameliorate ER stress–induced liver injury as well as HFD-induced metabolic disorders. Several previous reports have suggested that CO prevents HFD-induced obesity. However, the mechanisms for the preventive effects of CO on metabolic disorders remain unknown.

This study is the first, to our knowledge, to establish the critical role of FGF21 in the salutary effects of CO on metabolic disorders, using mice genetically deficient in FGF21. FGF21, as a potential therapeutic for metabolic disease, such as diabetes and obesity (51, 52), can increase thermogenesis. Specifically, FGF21 stimulates the browning of white fat in response to cold or stimulation with adrenergic agonists (25). Because FGF21 is linked with energy expenditure (25), thermogenesis increased by FGF21 may provide a mechanism for weight loss. Interestingly, CO-induced FGF21 expression enhanced the transcription of thermogenic genes. In mice genetically deficient in FGF21, CO failed to increase gene expression related to the browning of WAT. Therefore, we conclude that CO promotes increased thermogenic gene expression *via* induction of FGF21 expression.

In summary, CO protects against HFD-induced weight gain in wild-type *Fgf21^+/+^* mice. The changes in body weight induced by CO were accompanied by improved metabolic status *via* FGF21 induction. Thus, CO-induced FGF21 expression counteracts metabolic dysfunction in ER stress or HFD-induced pathologies through up-regulation of mitochondrial function and biogenesis, the decrease of white adipocyte size, and the browning WAT. Finally, CO activates the PERK-ATF4 pathway *via* enhanced ROS production, which enhances FGF21 expression.

Completed and ongoing clinical trials to date for CO therapy in humans have, in general, demonstrated the safety of low-dose CO application in humans, although further trials will be needed to confirm efficacy. It is, therefore, premature to speculate that CO will prove to be an effective therapeutic in humans for metabolic disease applications (53). Nevertheless, our findings that an increase of FGF21 by CO improves metabolic dysfunction suggest that a low-dose application of CO may represent a potential therapeutic strategy for the amelioration of metabolic diseases.

## Supplementary Material

This article includes supplemental data. Please visit *http://www.fasebj.org* to obtain this information.

## Abbreviations

ALT: alanine aminotransferase
AML: acute myeloid leukemia
AST: aspartate aminotransferase
ATF4: activating transcription factor 4
ATF6: activating transcription factor 6
ATGL: adipose triglyceride lipase
BAT: brown adipose tissue
CO: carbon monoxide
CORM: CO-releasing molecule
eIF2α: eukaryotic translation initiation factor 2α
ER: endoplasmic reticulum
eWAT: epididymal white adipose tissue
FGF21: fibroblast growth factor 21
GTT: glucose tolerance test
H&E: hematoxylin and eosin
HFD: high-fat diet
HSL: hormone-sensitive lipase
IRE1: inositol-requiring enzyme-1
ITT: insulin tolerance test
iWAT: inguinal white adipose tissue
MEF: mouse embryonic fibroblast
mtROS: mitochondrial reactive oxygen species
NAC: *N*-acetyl-l-cysteine
NCD: normal chow diet
OCR: oxygen consumption rate
PERK: protein kinase R-like endoplasmic reticulum kinase
Plin1: perilipin 1A
ppm: parts per million
qRT-PCR: quantitative RT-PCR
ROS: reactive oxygen species
scRNA: negative control siRNA
siRNA: small interfering RNA
TG: triglyceride
Tm: tunicamycin
WAT: white adipose tissue
