# Supplementary figures and images for: FGF21 induced by carbon monoxide mediates metabolic homeostasis via the PERK/ATF4 pathway
Source: FASEB J. 2018 Jan 8;32(5):2630–43. doi: 10.1096/fj.201700709RR (PMC5901375; doi:10.1096/fj.201700709RR)

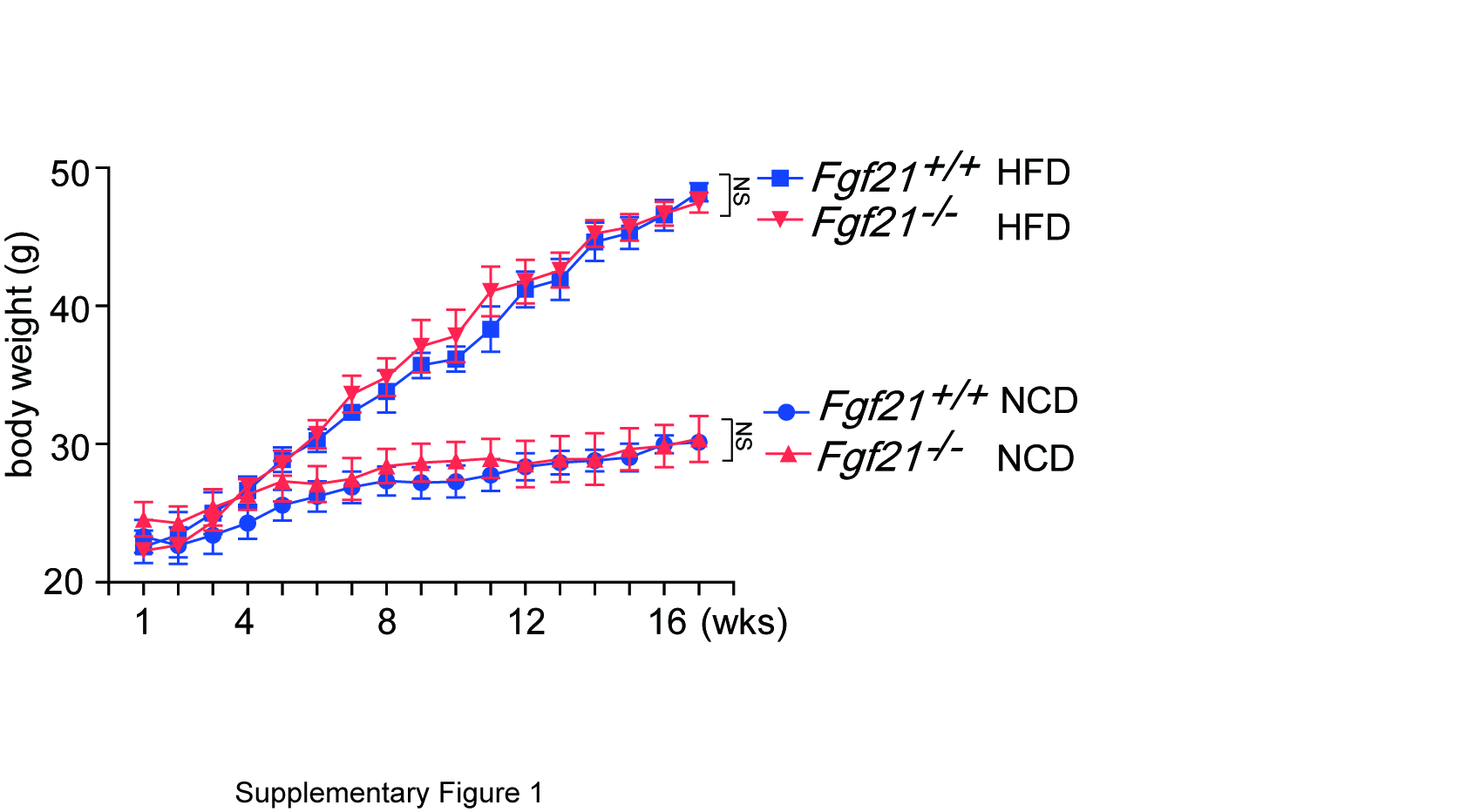

Supplement: Supplementary file 1 [file fj.201700709RR.sf1.tif]

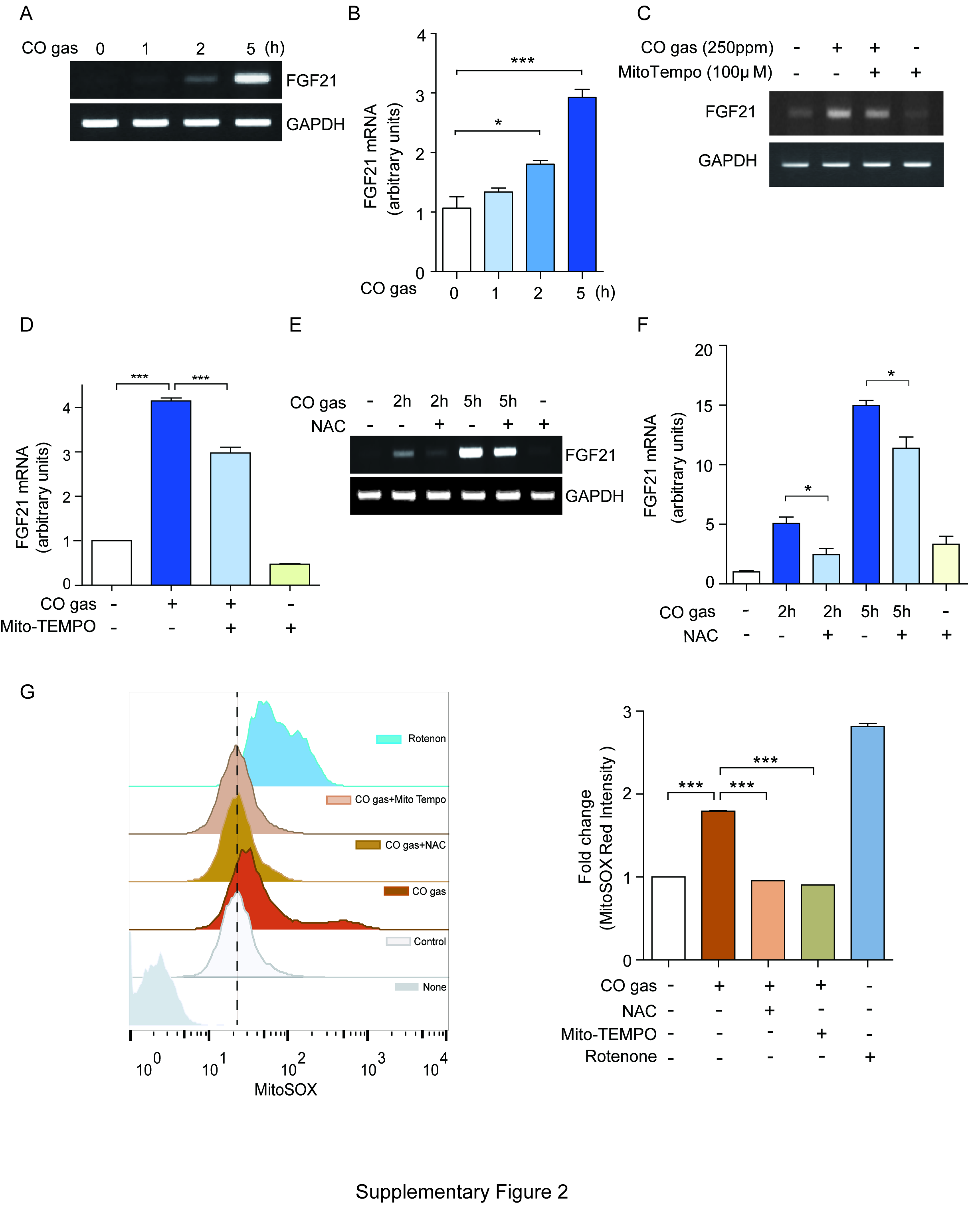

Supplement: Supplementary file 2 [file fj.201700709RR.sf2.tif]

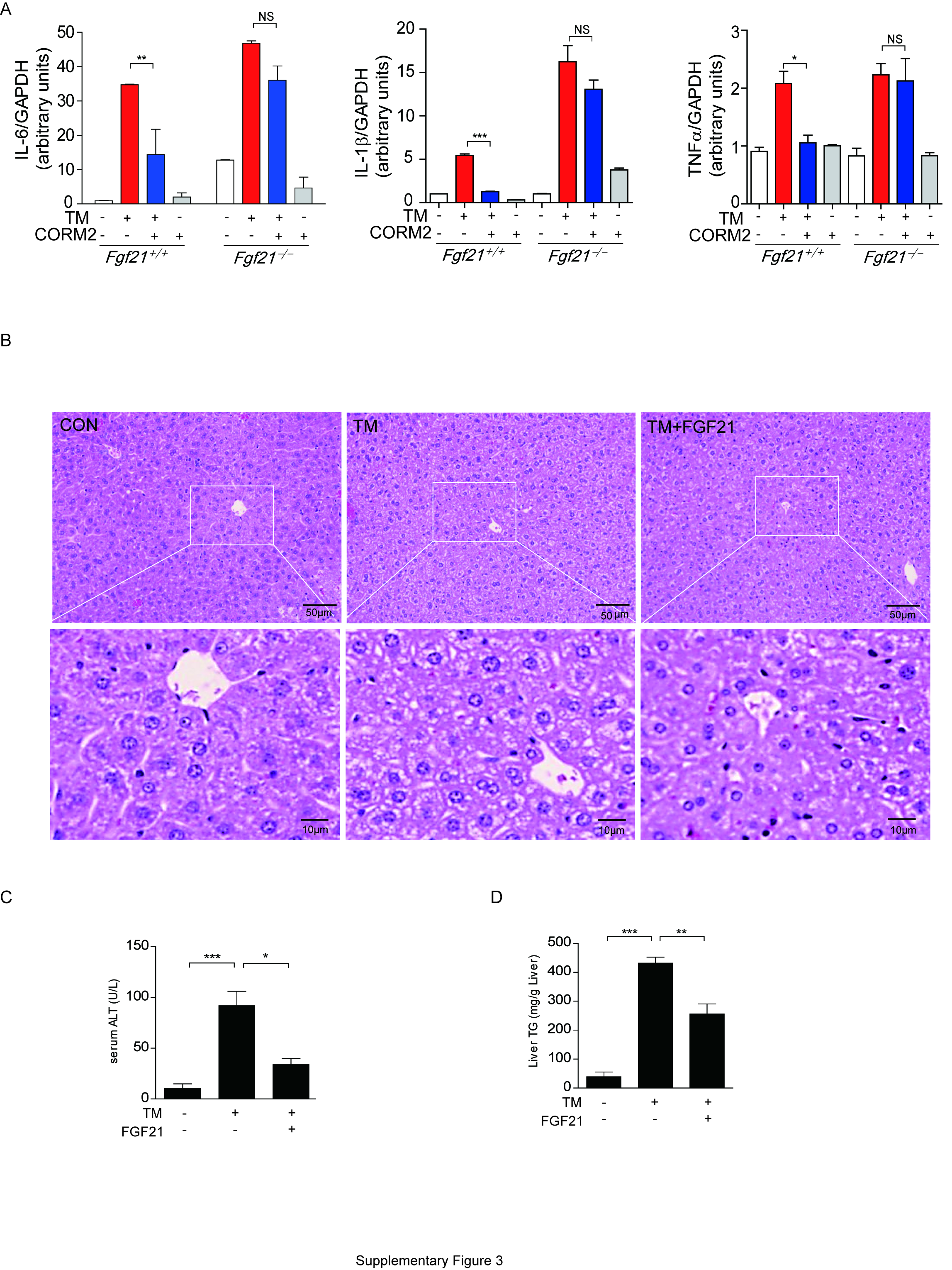

Supplement: Supplementary file 3 [file fj.201700709RR.sf3.tif]

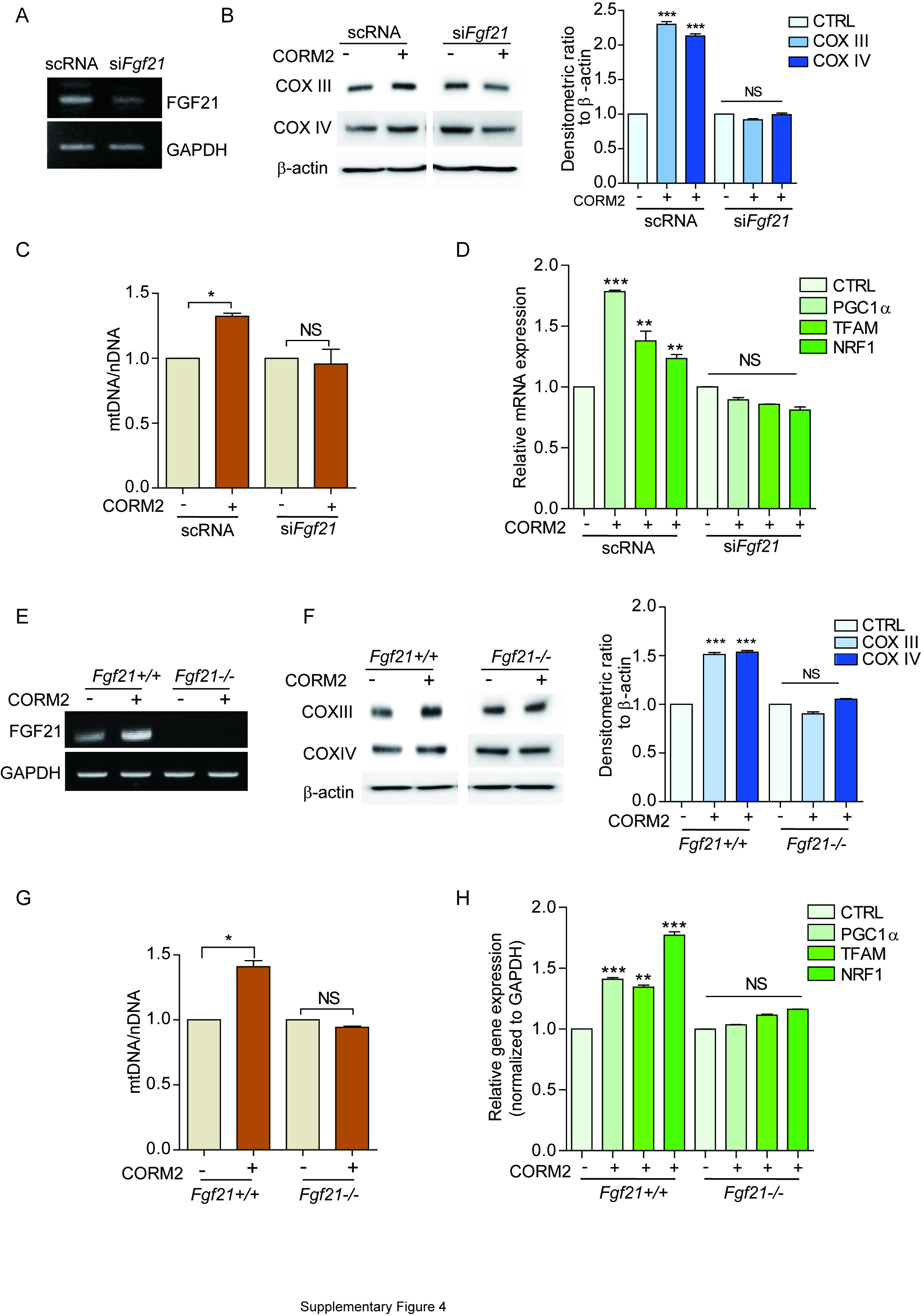

Supplement: Supplementary file 4 [file fj.201700709RR.sf4.tif]

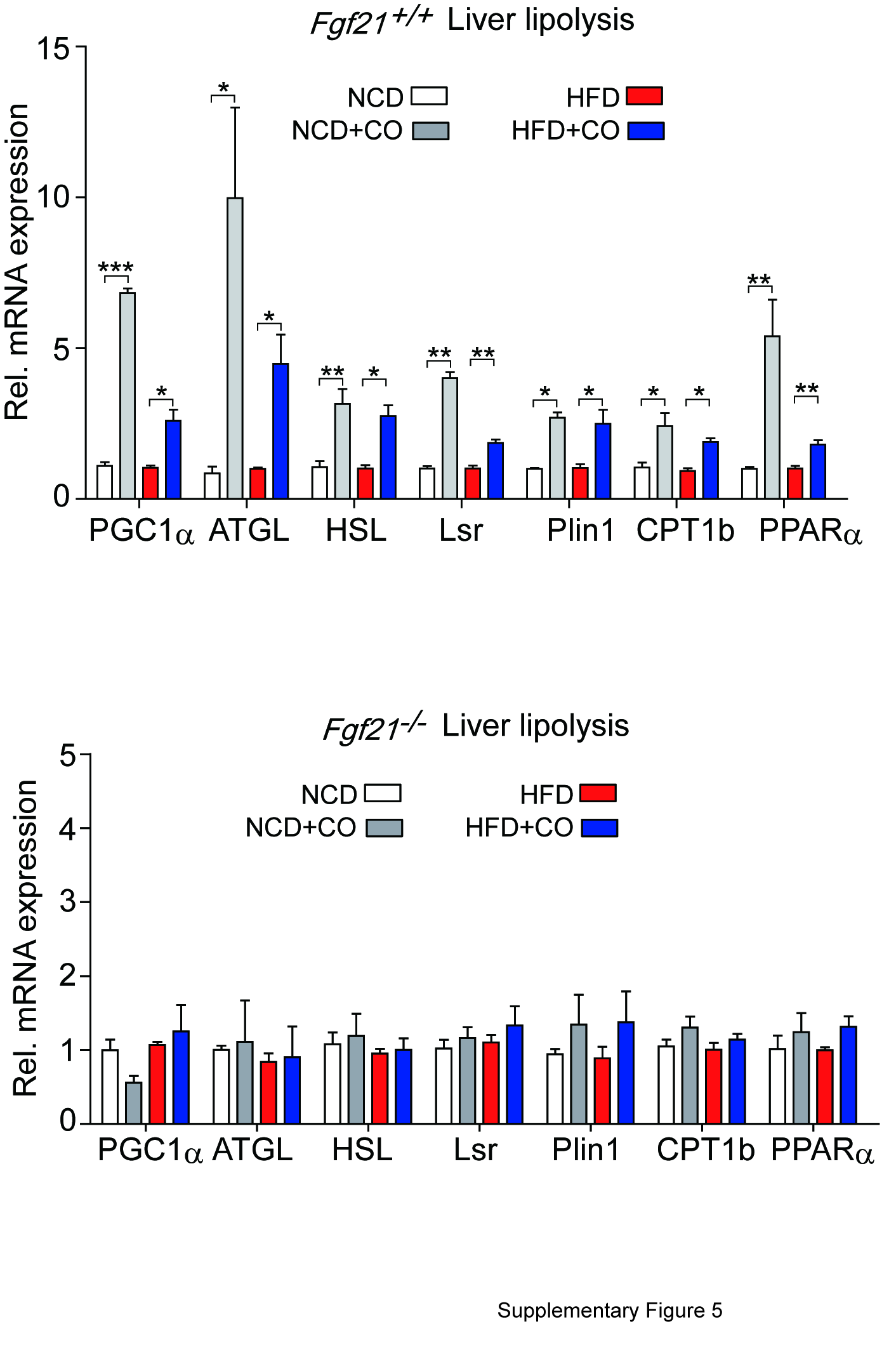

Supplement: Supplementary file 5 [file fj.201700709RR.sf5.tif]
