# Supplementary material for: FGF21 induced by carbon monoxide mediates metabolic homeostasis via the PERK/ATF4 pathway
Source: FASEB J. 2018 Jan 8;32(5):2630–43. doi: 10.1096/fj.201700709RR (PMC5901375; doi:10.1096/fj.201700709RR)
Supplement: Supplementary file 6 [file fj.201700709RR.st1.docx]

Supplementary Table 1. Gene primers for qRT-PCR

| **Gene** | **Forward primer 5’ to 3’** | **Reverse primer 5’ to 3’** |
| --- | --- | --- |
| mFGF21 | CAGGGGTCATTCAAATCCTG | GGAGTCCTTCTGAGGCAGAC |
| mCidea | ATCACAACTGGCCTGGTTACG | TACTACCCGGTGTCCATTTCT |
| mPRDM16 | CAGCACGGTGAAGCCATTC | GCGTGCATCCGCTTGTG |
| mUCP1 | ACTGCCACACCTCCAGTCATT | CTTTGCCTCACTCAGGATTGG |
| mPGC1α | AGCCGTGACCACTGACAACGAG | GCTGCATGGTTCTGAGTGCTAAG |
| mLsr | CAACCGGCCTGGCTCCACTG | AGGTCATCCCGGCTGCGACT |
| mATGL | GCCACAGCGCTGGTCACT | CCTCCTTGGACACCTCAATAATG |
| mHSL | AGGCCTCAGTGT GACCGCCA | GCCCCACGCAACTCTGGGTC |
| mPlin1 | TGGCCTCTGGAGGGGCTGAT | GGCCTTGGGAGCCTTCTGGG |
| mCPT1b | ACCGTGAAGAGATCAAGCCG | TCTCTTTGCCTGGGATGCGT |
| mPPARα | AGAGCCCCATCTGTCCTCTC | ACTGGTAGTCTGCAAAACCA |
| mPPARγ | TGTGGGGATAAAGCATCAGG | CCGGCAGTTAAGATCACACC |
| mGAPDH | GGGAAGCCCATCACCATCT | CGGCCTCACCCCATTTG |
| mNRF-1 | CGCAGCACCTTTGGAGAA | CCCGACCTGTGGAATACTTG |
| mTFAM | GGAATGTGGAGCGTGCTAAAA | TGCTGGAAAAACACTTCGGAATA |
