# Supplementary material for: FGF21 induced by carbon monoxide mediates metabolic homeostasis via the PERK/ATF4 pathway
Source: FASEB J. 2018 Jan 8;32(5):2630–43. doi: 10.1096/fj.201700709RR (PMC5901375; doi:10.1096/fj.201700709RR)
Supplement: Supplementary file 7 [file fj.201700709RR.sd1.docx]

**Supplementary Information**

**FGF21 Induced by Carbon Monoxide Mediates Metabolic Homeostasis *via* the PERK/ATF4 Pathway**

Yeonsoo Joe^*,1^, Sena Kim^*,1^, Hyo Jeong Kim^*^, Jeongmin Park^*^, Yingqing Chen^*^, Hyeok-Jun Park^*^, Seung-Joo Jekal ^†^, Stefan W. Ryter^‡^, Uh Hyun Kim^§,2^, and Hun Taeg Chung^*,2^

^*^School of Biological Sciences, University of Ulsan, Ulsan, 44610, Korea, ^†^Wonkwang Health Science University, Iksan, Jeonbuk, 54538, Korea, ^‡^Joan and Sanford I. Weill Department of Medicine, and Division of Pulmonary and Critical Care Medicine, Weill Cornell Medicine, New York, NY 10065, USA, ^§^National Creative Research Laboratory for Ca^2+^ signaling Network, Chonbuk National University Medical School, Jeonju, 54907, Korea,

**Supplementary Figure 1.**  6-week-old male *Fgf21^+/+^* and *Fgf21^-/-^* mice (n=6 mice/ group) were fed an NCD or HFD for 16 weeks. The average body weight was measured every two days. NS, not significant.

**Supplementary Figure 2.** **(A and B)** Primary hepatocytes were treated with CO gas (250 ppm) for various times (0, 1, 2 and 5 hrs), and then the mRNA level of FGF21 was determined by RT-PCR (A) and qRT-PCR (B). **(C and D)** AML12 cells were pretreated with Mito-TEMPO (100 μM) for one hr followed by the administration of CO gas (250 ppm) for another 2 hrs. FGF21 mRNA expression was determined by RT-PCR (C) and qRT-PCR (D). **(E and F)** AML12 cells were pretreated with NAC (3 mM) for 30 min followed by exposure to CO gas (250 ppm) for another 2 and 5 hrs. FGF21 mRNA levels were measured by RT-PCR (E) and qRT-PCR (F). To measure mtROS, AML12 cells were pretreated with NAC (3 mM) for 30 min and Mito-TEMPO (100 μM) for one hr, and then were exposed to CO gas (250 ppm) for another 2 hours. The production of mtROS was detected with MitoSOX Red (5 μM) for 50 min, and measured by flow cytometry. Rotenone (10 μM) was used as a positive control for mtROS production. Data were presented as mean±SEM (n=3). *, P<0.05; **, P<0.01; ***, P<0.001; NS, statistically not significant**.**

**Supplementary Figure 3.** (A) The mRNA expression of inflammatory cytokines IL-6, IL-1β and TNFα were detected in liver tissues extracted from *Fgf21^+/+^* and *Fgf21^-/-^* mice after 24-hr stimulation with TM. (B) 6-week-old C57BL/6 mice were pretreated with recombinant FGF21 (i.p., 0.5 mg/kg) for 24 hrs followed by challenge with TM (1 mg/kg, i.p.). At 24 hrs, Hematoxylin and eosin (H&E) staining was performed in liver tissues. Additionally, serum ALT (C) and liver TG (D) levels were also measured. Data were presented as mean±SEM (n=3). *, P<0.05; **, P<0.01; ***, P<0.001; NS, statistically not significant.

**Supplementary Figure 4**. (A) AML12 cells were transfected with scRNA or siRNA against *Fgf21* for 36 hrs, and the mRNA expression of FGF21 was measured by reverse transcriptase (RT)-PCR. (B) After the transfection, AML12 cells were treated with CORM2 (20 μM) for 18 hrs, and protein levels of COX III and COX IV were detected by Western blot (*left panel*). The bar graph (*right panel*) represents the densitometric ratio of COX III and COX IV to β-actin. (C) The relative mitochondrial DNA (mtDNA) content was measured by real-time (qRT)-PCR. The mtDNA content was normalized by nuclear DNA (nDNA) content. (D) mRNA levels of PGC1α, TFAM and NRF1 were also assessed by qRT-PCR after treatment with CORM2. (E) Primary hepatocytes extracted from *Fgf21^+/+^* and *Fgf21^-/-^* mice were treated with CORM2 (20 μM) for 18 hrs, and the mRNA expression of FGF21 was determined by RT-PCR. (F) The protein levels of COX III and COX IV were detected by Western blot (*left panel).* The bar graph (*right panel*) represents the densitometric ratio of COX III and COX IV to β-actin. (G) The ratio of mtDNA and nDNA was also determined after the administration of CORM2 in primary hepatocytes. (H) The mRNA levels of PGC1α, TFAM and NRF1 were detected by RT-PCR, and the expression of these genes was normalized by GAPDH. The bar graph represents the densitometric ratio of the respective mRNA to GAPDH. Data were presented as mean±SEM (n=3). *, *P*<0.05; **, *P*<0.01; ***, *P*<0.001; NS, not significant.

**Supplementary Figure 5.** The mRNA expression of genes (PGC1α, ATGL, HSL, Lsr, Plin1, CPT1b and PPARα) associated with lipolysis, were detected by qRT-PCR in liver tissues from *Fgf21^+/+^* and *Fgf21^-/-^* mice. *, P<0.05; **, P<0.01; ***, P<0.001; NS, statistically not significant.
